# Computed tomography assessment of the gastric arterial anatomy for embolisation treatment of obesity

**DOI:** 10.1186/s42155-025-00552-z

**Published:** 2025-10-16

**Authors:** Richard Lindsay Hesketh, Rayhab Mashal, Natasha Thorley, Jowad Raja, Julian Hague, Robert Thomas, Prashant Patel, Ahmed Ahmed, Mohamad Hamady

**Affiliations:** 1 Centre for Medical Imaging, University College London, 43 - 45 Foley Street, London, W1 W 7 TS UK; 2 Department of Interventional Radiology, University College London Hospital, 235 Euston Road, London, NW1 2BU UK; 3 Department of Interventional Radiology, Imperial College Healthcare NHS Trust, London, UK; 4 Department of Surgery, Imperial College Healthcare NHS Trust, London, UK; 5 Department of Cancer and Surgery, Imperial College, London, UK; 6 Department of Radiology, University College London Hospital, 235 Euston Road, London, NW1 2BU UK

## Abstract

**Background:**

Obesity is a global pandemic affecting more than 1 billion people worldwide and a leading cause of preventable death. Left gastric artery embolisation to inhibit ghrelin secretion, a hormonal driver of appetite, has been proposed as a potentially safer treatment for obesity than surgery. This study describes the incidence of anatomical variation, gastric artery anastomoses, collateral supply and vessel lengths, diameters and angles of origin relevant to embolisation of the LGA in the EMBIO cohort of obese patients.

**Results:**

Arterial phase CT scans (*n* = 90) were performed as part of screening for the EMBIO trial. 62 participants (69%) had conventional coeliac and hepatic artery anatomy. 14 participants (16%) had left hepatic arterial supply originating from the LGA. The most common LGA branching pattern (37%) was for the first branch to supply the cardia / gastro-oesophageal junction and then for the LGA to split into two main branches supplying the gastric fundus. 34% had a left- right gastric artery anastomosis visible on CT. The LGA was the dominant artery supplying the gastric fundus in 51% with supply from multiple arteries, most frequently the LGA and short gastric arteries (27%), seen in the other participants.

**Conclusion:**

This study presents detailed analysis of the arterial anatomy relevant to performing successful and safe embolisation of the LGA for treatment of obesity and acute haemorrhage.

**Graphical Abstract:**

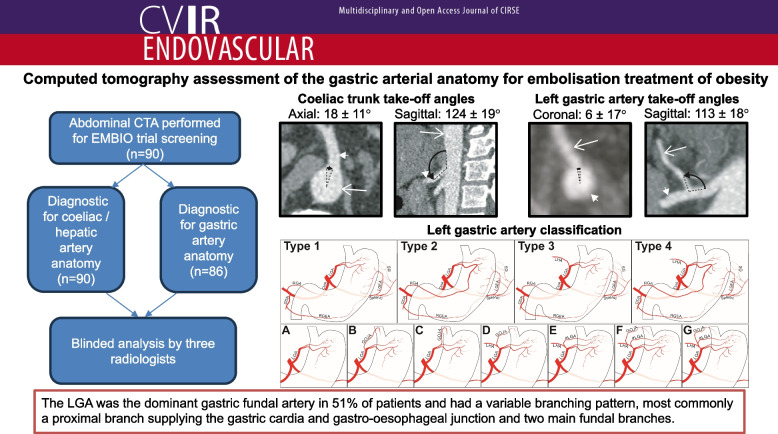

**Supplementary Information:**

The online version contains supplementary material available at 10.1186/s42155-025-00552-z.

## Background

Obesity is a pandemic affecting more than 1 billion people worldwide and a leading cause of preventable death with individuals at increased risk of type II diabetes, cardiovascular disease and cancer [1]. Even modest reductions in weight of 5–10% improves metabolic function and reduces the associated morbidity [2, 3]. Bariatric surgery typically achieves 20–30% of sustained weight loss but has rare but severe potential complications and surgical eligibility significantly outstrips capacity in most countries [4–7]. Alternative treatments for weight loss are diet and lifestyle therapies and pharmacological treatments, but the sustained weight loss associated with these is less than < 5% [8]. GLP- 1 agonists e.g., semaglutide, have improved short-term efficacy but are expensive, long-term safety has not been demonstrated and weight loss is largely reversed after cessation of treatment [9].

Embolisation of the gastric blood supply has been proposed as a potentially safer and more cost-effective alternative for weight loss treatment avoiding the risks of surgery and side effects of medication. The left gastric artery (LGA) primarily supplies the gastric fundus and embolisation is thought to cause weight loss by suppressing ghrelin production from fundal P/D1 cells [10]. Although long term benefits are still unknown, preliminary cohort studies have shown promise for this technique in humans [11, 12]. Therefore, a thorough understanding of the gastric blood supply is essential for interventional radiologists, for both the management of gastrointestinal bleeding and potential future application in the management of obesity. This cross-sectional study reports the relevant arterial anatomy from computed tomography (CT) and catheter angiography of participants enrolled in the Embolisation in Obesity (EMBIO) double-blinded, placebo-controlled randomised controlled trial of left gastric artery embolisation to provide a unique insight into gastric arterial anatomy [13]. If positive, the trial would pave the way for embolisation becoming part of the bariatric treatment algorithm.

## Methods

### Participants

Participants in both arms of the EMBIO trial were included [13]. Ninety-six patients were screened for inclusion in the EMBIO trial between June 2022 and July 2023. Six participants were excluded because CT images were unavailable (either not performed due to prior screening failure or participant withdrawal). Ninety participants were included, comprising fourteen males and seventy-six females with a mean age of 49 ± 13 years.

### Computed tomography

Helical, arterial phase CT scans of the upper abdomen were acquired in inspiration using Ingenuity (Philips, The Netherlands) and Aquilion Prime (Canon Medical Systems, Japan) scanners following injection of 80–100 mL of iohexol (Omnipaque 300, GE Healthcare, IL, USA). Studies were reconstructed in 3D with a slice thickness of 0.5 - 1 mm.

### Catheter angiography

Catheter angiograms were performed by hand injection of iohexol into the coeliac trunk or LGA in participants assigned to the treatment arm of the trial.

### Image analysis

Three board-certified radiologists with five – seven years experience in vascular radiology performed blinded analysis using VuePACS (Philips, The Netherlands), with each scan being assessed by at least two radiologists. Image window width and level for analysis was at the readers discretion. Measurements of aortic, coeliac trunk and LGA vessel diameter and angles of coeliac and LGA origin were made (Tables 1 and 2). Gastric arterial anatomy was assessed and classified on CT, with catheter angiograms used to illustrate the anatomy.

**Table 1:** Location of vessel diameter measurements

**Vessel**	**Location**	**Measurements**	**CT Reconstruction**
Abdominal aorta (Fig. 2)	3 cm above the coeliac trunk ostium	AP and TR diameter	Minimum slice thickness
Abdominal aorta (Fig. 1).	Level of the coeliac trunk ostium	AP and TR diameter	Minimum slice thickness
Coeliac trunk (Fig. 4)	1 cm downstream from the ostium	AP and TR diameter	Minimum slice thickness
LGA	1 cm downstream from the ostium	AP and TR diameter	10 mm MIP

*Abbreviations*: *AP* anterior-posterior plane, *LGA *left gastric artery, *MIP *maximum intensity projection, *TR *transverse plane

**Table 2:** Location and methods of angle measurement. Angles were measured using multi-parametric reconstruction (MPR) to align in plane with the parent vessel for para-sagittal measurements or perpendicular to the vessel for clockwise measurements

**Vessel angle**	**CT orientation/angle measured**	**Angle measurement method**
Coeliac trunk from the aorta (Fig. 1)	Axial (clockwise)	Point 1: 12 o’clock (anteriorly) Point 2/vertex: centre of the aorta Point 3: middle of the coeliac trunk at the ostium
Coeliac trunk from the aorta (Fig. 2)	Para-sagittal	Point 1: anterior wall of the aorta 1 cm superior to the coeliac trunk Point 2/vertex: centre of the coeliac trunk ostium Point 3: centre of the coeliac trunk 1 cm downstream of the ostium
LGA from the coeliac trunk (Fig. 4)	Para-coronal (clockwise)	Point 1: 12 o’clock (superiorly) Point 2/vertex: centre of the coeliac trunk Point 3: centre of the LGA ostium
LGA from the coeliac trunk (Fig. 3)	Para-sagittal	Point 1: superior border of the coeliac trunk 1 cm upstream of the LGA ostium Point 2/vertex: centre of the LGA ostium Point 3: centre of the LGA 1 cm downstream of the ostium

### Statistical analysis

Statistical and graphical analysis was performed using Matlab v5 (Mathworks, MA, USA) and Prism v6 (Graphpad, CA, USA). Distributions were tested for normality using D’Agostino & Pearson omnibus normality tests and significant difference between the means tested using paired t-tests and Wilcoxon matched-pairs signed rank tests for normally distributed and skewed data, respectively [14]. Bland-Altman plots were used to assess inter-reader agreement and bias [15].

## Results

The diagnostic quality of scans was sufficient to assess the anatomy of the aorta, coeliac trunk and its 1^st^ order branches in all participants and the branching anatomy of the LGA eighty-six participants (96%).

### Thoracoabdominal aorta

The thoracoabdominal aorta had an ellipsoid cross-sectional shape longer in the transverse (TR) than the anterior-posterior (AP) axis (*P* < 0.0001; *n* = 90). Craniocaudally it had an inverted conical shape, reducing from 21.5 ± 2.7 (95% CI: 20.9–22.0) (TR) and 20.2 ± 2.5 (95% CI: 19.7–20.8) (AP) 3 cm above the level of the coeliac trunk to 20.3 ± 2.4 cm (95% CI: 19.8–20.8) (TR) and 19.4 ± 2.5 (95% CI: 18.9–20.0) (AP) at the level of the coeliac trunk.

### Coeliac and hepatic arterial anatomy

**Fig. 1:**
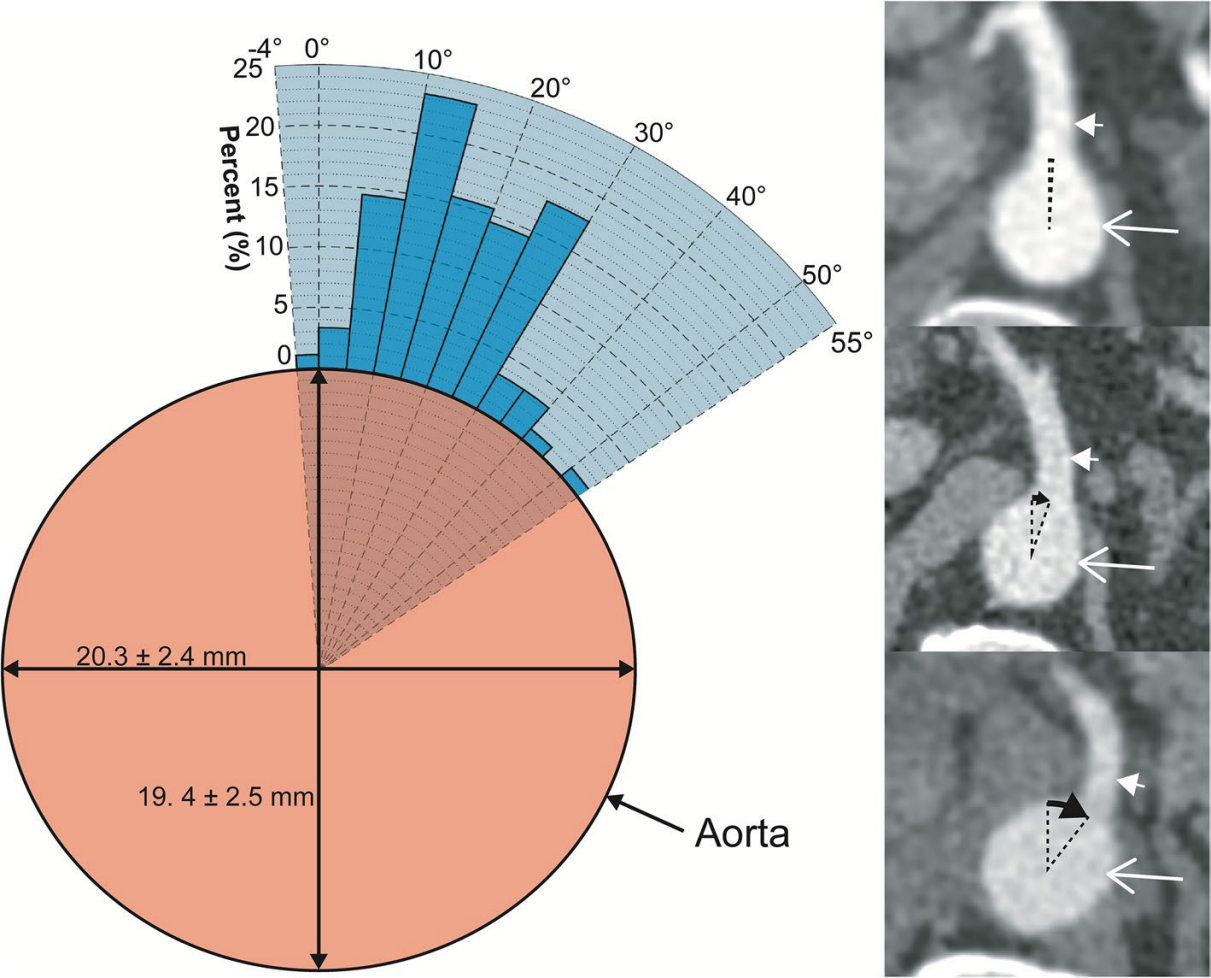
Clockwise angle of the origin of the coeliac trunk from the aorta in the axial plane. Histogram of the angles of origin of coeliac trunk from the aorta in the axial plane plotted as a percentage of participants. The minimum and maximum angle is represented by the radial range of the graph (− 4 to + 55°; light blue shading). The cross-sectional dimensions of the aorta at the level of the coeliac trunk are also included. Inset CT images show examples and calculations for the coeliac trunk clockwise angle from participants in the (top to bottom) lowest, median and highest decile. Long white arrow, aorta; short white arrow, coeliac trunk; black arrow, measured angle

**Fig. 2:**
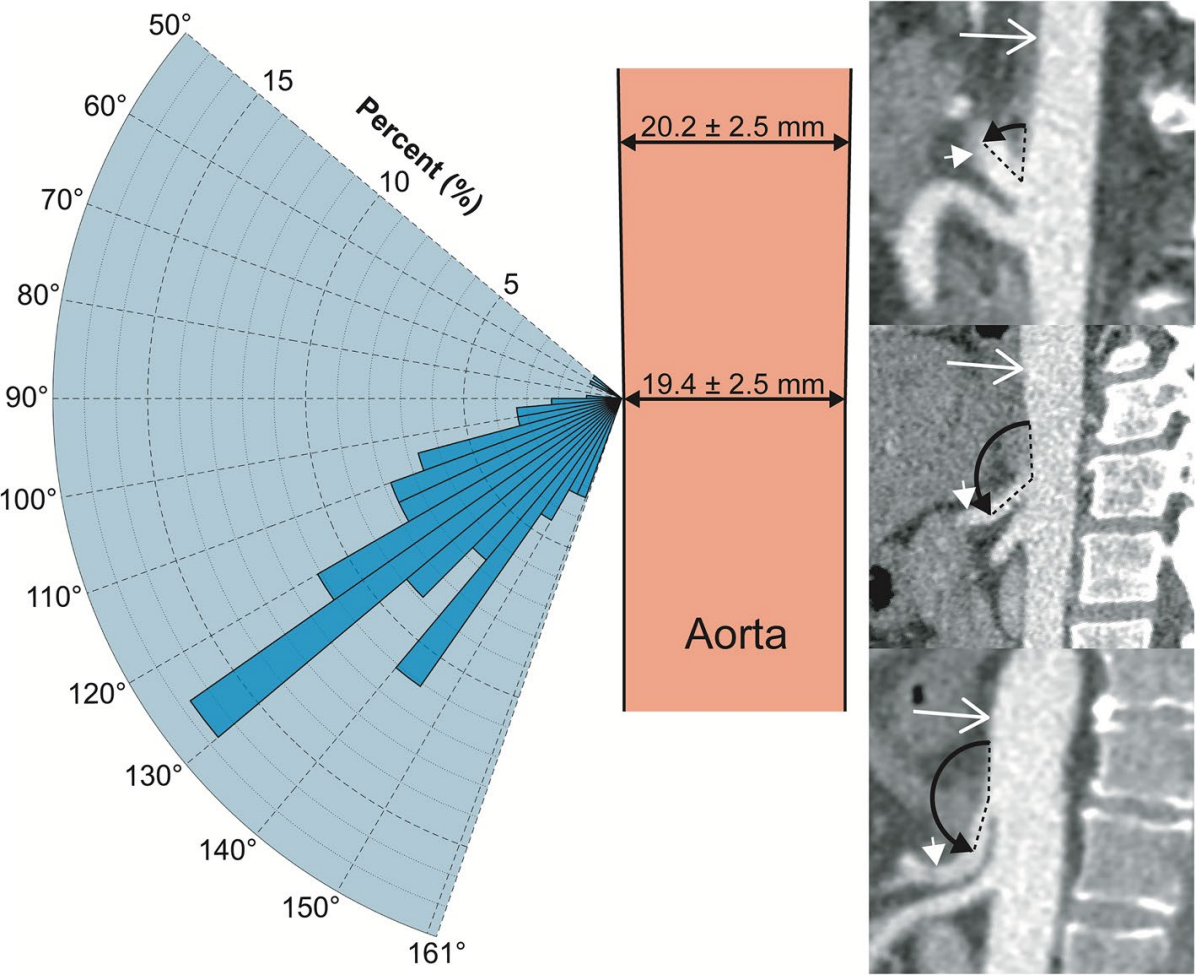
Angle of the origin of the coeliac trunk from the aorta in the para-sagittal plane**.** Histogram of the angle of origin of coeliac trunk from the aorta in the para-sagittal plane plotted as a percentage of participants. The minimum and maximum angle is represented by the radial range of the graph (50 to 161°). The anterior-posterior dimensions of the aorta at the level of the coeliac trunk are also included. Inset CT images show examples and calculations for the coeliac trunk para-sagittal angle from participants in the (top to bottom) lowest, median and highest decile. Long white arrow, aorta; short white arrow, coeliac trunk; black arrow, measured angle

The ostium of the coeliac trunk from the aorta was located between the T12 and L1 vertebral level in all participants and in the axial plane at a clockwise angle of 18.3 ± 10.5° where 0° was located at an anterior 12 o’clock position (Fig. 1). In the para-sagittal plane the coeliac trunk had an angle of 124.4 ± 19.0° from the aorta (Fig. 2). The coeliac trunk had a mean diameter of 7.0 ± 0.5 mm.

Sixty-two participants (69%) had conventional coeliac and hepatic anatomy (Tables 3 and 4). Fourteen participants (16%) had left hepatic supply from the LGA, twelve of whom had an accessory left hepatic artery from the LGA limited to supplying segments II and III and two that had a fully replaced left hepatic artery from the LGA supplying the whole left lobe. The LGA was diminutive in two participants both of whom either had an accessory left hepatic artery or fully replaced left hepatic artery.

**Table 3:** Coeliac and hepatic artery anatomy

	**Coeliac Trunk Anatomy**						
**Hepatic Artery Anatomy**	**Normal**	**Gastrosplenic trunk**	**Hepatosplenic trunk**	**Coeliacomesenteric trunk**	**Quadfurcation of coeliac trunk**	**Splenomesenteric trunk**	**Total**
**Normal**	62		2	1			**65**
**aLHA from the LGA**	8		1	1			**10**
**Replaced RHA from the SMA**	3					1	**4**
**CHA from the aorta**		3					**3**
**CHA from the SMA**		2					**2**
**Replaced RHA from the CTr**					2		**2**
**Replaced LHA from the LGA**	2						**2**
**aRHA from the CTr**	1						**1**
**Replaced RHA from the SMA and aLHA originating from the LGA**	1						**1**
**Total**	**77**	**5**	**3**	**2**	**2**	**1**	**90**

*Abbreviations*: *aLHA* accessory left hepatic artery, *aRHA* accessory right hepatic artery, *CHA* common hepatic artery, *CTr* coeliac trunk, *LGA* left gastric artery, *LHA* left hepatic artery, *RHA* right hepatic artery, *SMA* superior mesenteric artery, *U* unclassified

**Table 4:** Classification of coeliac (Uflacker) and hepatic artery (Michels and Hiatt) anatomy

**Coeliac Trunk**		**Hepatic Artery**			
**Uflacker Classification**	**Number (%)**	**Michels Classification**	**Number (%)**	**Hiatt Classificaiton**	**Number (%)**
I (conventional)	77 (86)	I (conventional)	65 (72)	I (conventional)	65 (72)
II (hepatosplenic trunk)	3 (3)	II (replaced LHA from the LGA)	2 (2)	II (replaced LHA or aLHA from the LGA)	12 (13)
V (gastrosplenic trunk)	5 (6)	III (replaced RHA from the SMA)	4 (4)	III (replaced RHA or aRHA from the SMA)	4 (4)
VI (coeliacomesenteric trunk)	2 (2)	V (aLHA from the LGA)	10 (11)	IV (II + III)	1 (1)
Unclassified	3 (3)	VIII (aLHA from LGA and RHA from the SMA)	1 (1)	V (CHA from the SMA)	2 (2)
		IX (CHA from the SMA)	2 (2)	VI (CHA from the aorta)	3 (3)
		Unclassified	6 (7)	Unclassified	3 (3)
Total	90	Total	90	Total	90

*Abbreviations: aLHA* accessory left hepatic artery, *aRHA* accessory right hepatic artery, *CHA* common hepatic artery, *CTr* coeliac trunk, *LGA* left gastric artery, *LHA* left hepatic artery, *RHA* right hepatic artery, *SMA* superior mesenteric artery, *U* unclassified

### Left gastric arterial anatomy

**Fig. 3:**
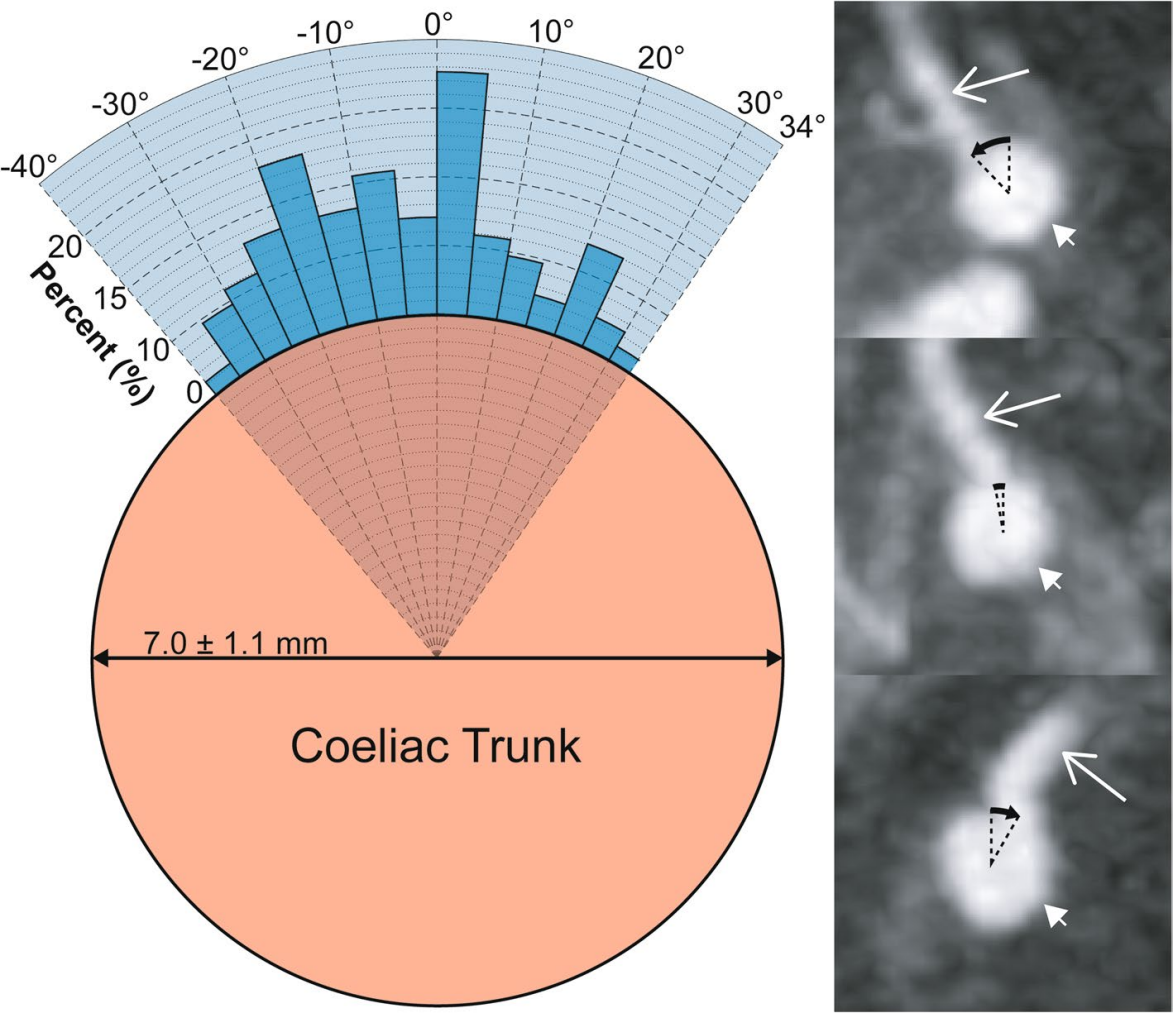
Clockwise angle of the origin of the LGA from the coeliac trunk in the para-coronal plane. Histogram of the clockwise angle of origin of LGA in a para-coronal plane through the coeliac trunk plotted as a percentage of participants. The minimum and maximum angle is represented by the radial range of the graph (− 40 to + 34°). The cross-sectional dimensions of the coeliac trunk are also included. Inset CT images show examples and calculations for the LGA clockwise angle from participants in the (top to bottom) lowest, median and highest decile. Long white arrow, LGA; short white arrow, coeliac trunk; black arrow, measured angle

**Fig. 4:**
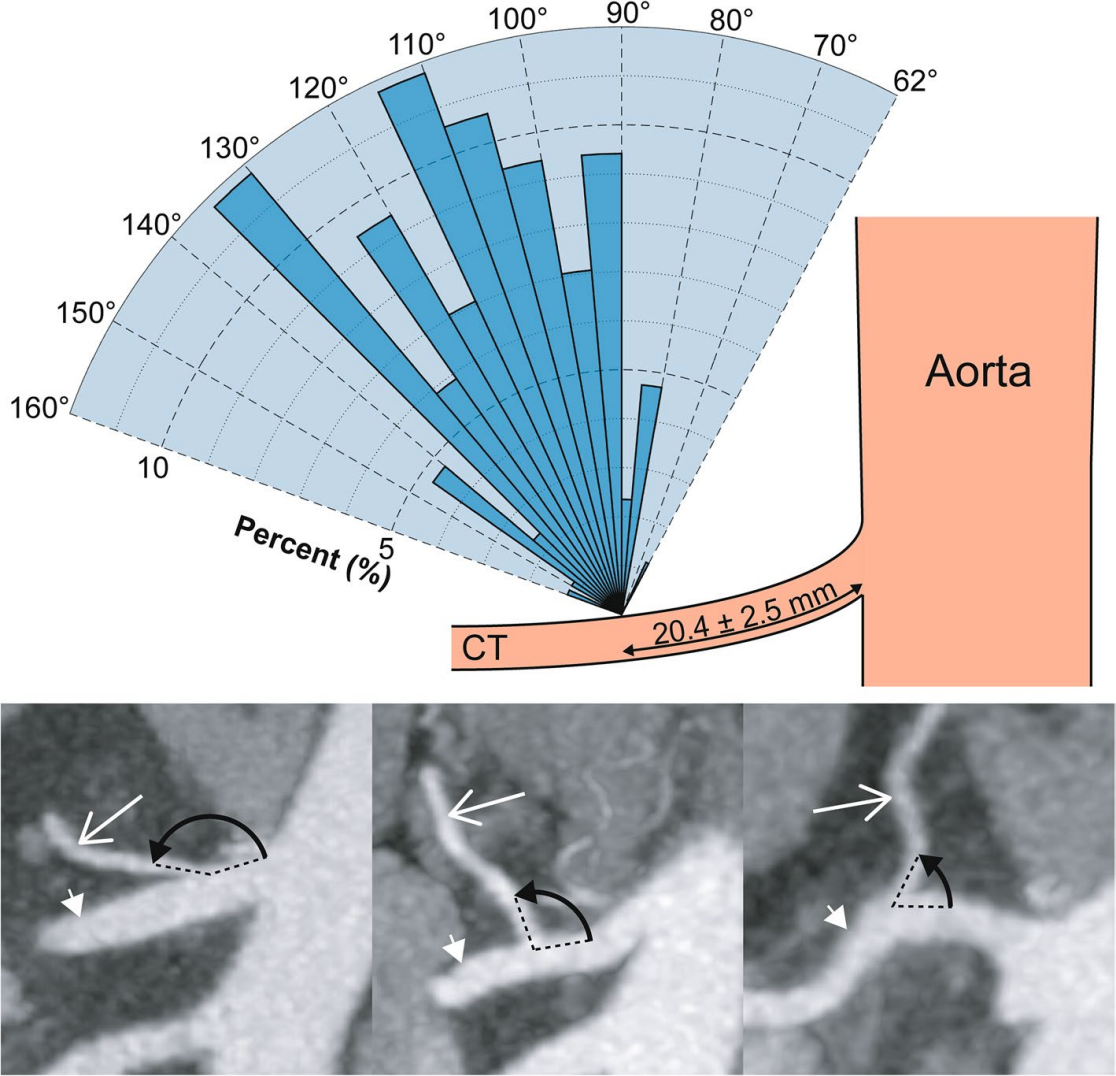
Angle of the origin of the LGA from the coeliac/coeliacomesenteric/gastrosplenic trunk in the para-sagittal plane. Histogram of the angle of origin of LGA from the parent vessel plotted as a percentage of participants. The minimum and maximum angle is represented by the radial range of the graph (62 to 160°). Inset CT images show examples and calculations for the LGA clockwise angle from participants in the (left to right) highest, median and lowest decile. Long white arrow, LGA; short white arrow, coeliac trunk; black arrow, measured angle

When arising from the coeliac, coeliacomesenteric or gastrosplenic trunk the LGA ostium was located 20.4 ± 6.7 mm from the aorta, originated at a para-sagittal angle of 112.6 ± 17.9° from the parent vessel and at a para-coronal clockwise angle of − 6.4 ± 16.8° where 0° was located at a cranial 12 o’clock position (Figs. 3 and  4). The diameter of the LGA was 2.8 ± 0.6 mm.

Four participants (4%) had a hepatosplenic trunk in which the LGA originating directly from the aorta just superior to the hepatosplenic trunk at a clockwise (axial) angle of 6.3 ± 5.7° and a para-sagittal angle of 131 ± 17.0°.

### Inferior phrenic arteries

The inferior phrenic arteries can be inadvertently catheterised when trying to access the LGA and the left inferior phrenic artery can take a similar course to the LGA on anterior – posterior projection angiography (Fig. S3). The right inferior phrenic artery origin was identified with consensus between readers in 72 out of 90 participants and arose from the coeliac trunk (47%), aorta (39%), LGA (7%), right renal artery (6%) and common hepatic artery (1%). The left inferior phrenic artery origin was identified with consensus in 76 out of 90 participants, arising from the coeliac trunk (59%); aorta (37%); gastrosplenic trunk (1%); LGA (1%) and accessory left hepatic artery (1%). When originating from the coeliac trunk or LGA the inferior phrenic arteries were invariably the first branch of that artery.

### Branching pattern of the LGA

**Fig. 5:**
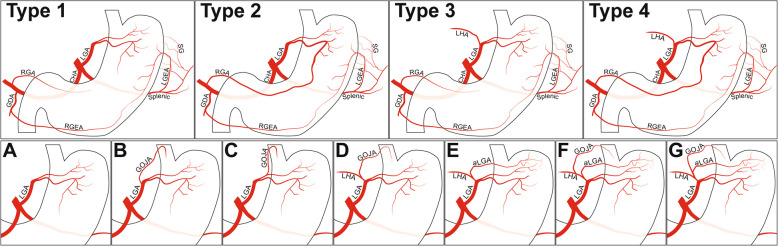
Diagrams of left gastric artery classification. **1** LGA supplying the fundus with no collateral supply demonstrated; **2** Visible LGA anastomosis with the RGA; **3** left hepatic supply from the LGA, either an accessory LHA or a replaced LHA; **4** both left hepatic supply and RGA anastomosis from the LGA (a combination of (**3**) and (**4**)). LGA subclassification depending on the anatomy of a gastro-oesophageal artery (GOJA) supplying the gastric cardia/gastro-oesophageal junction/inferior oesophagus and the accessory LGA. Subtypes: (**A**) No GOJA from the LGA; **B** GOJA arises from the main LGA; **C** GOJA from the superior LGA branch; **D** GOJA from the LHA; **E** accessory LGA from the LHA; **F** separate accessory LGA and GOJA from the LHA; **G** GOJA from an accessory LGA. Abbreviations: LGA, left gastric artery; GOJA, gastro-oesophageal artery; LHA, accessory or replaced left hepatic artery; aLGA, accessory left gastric artery; GDA, gastro-duodenal artery; RGA, right gastric artery; CHA, common hepatic artery; LGEA; left gastro-epiploic artery; RGEA, right gastro-epiploic artery; SG, short gastric arteries

**Fig. 6:**
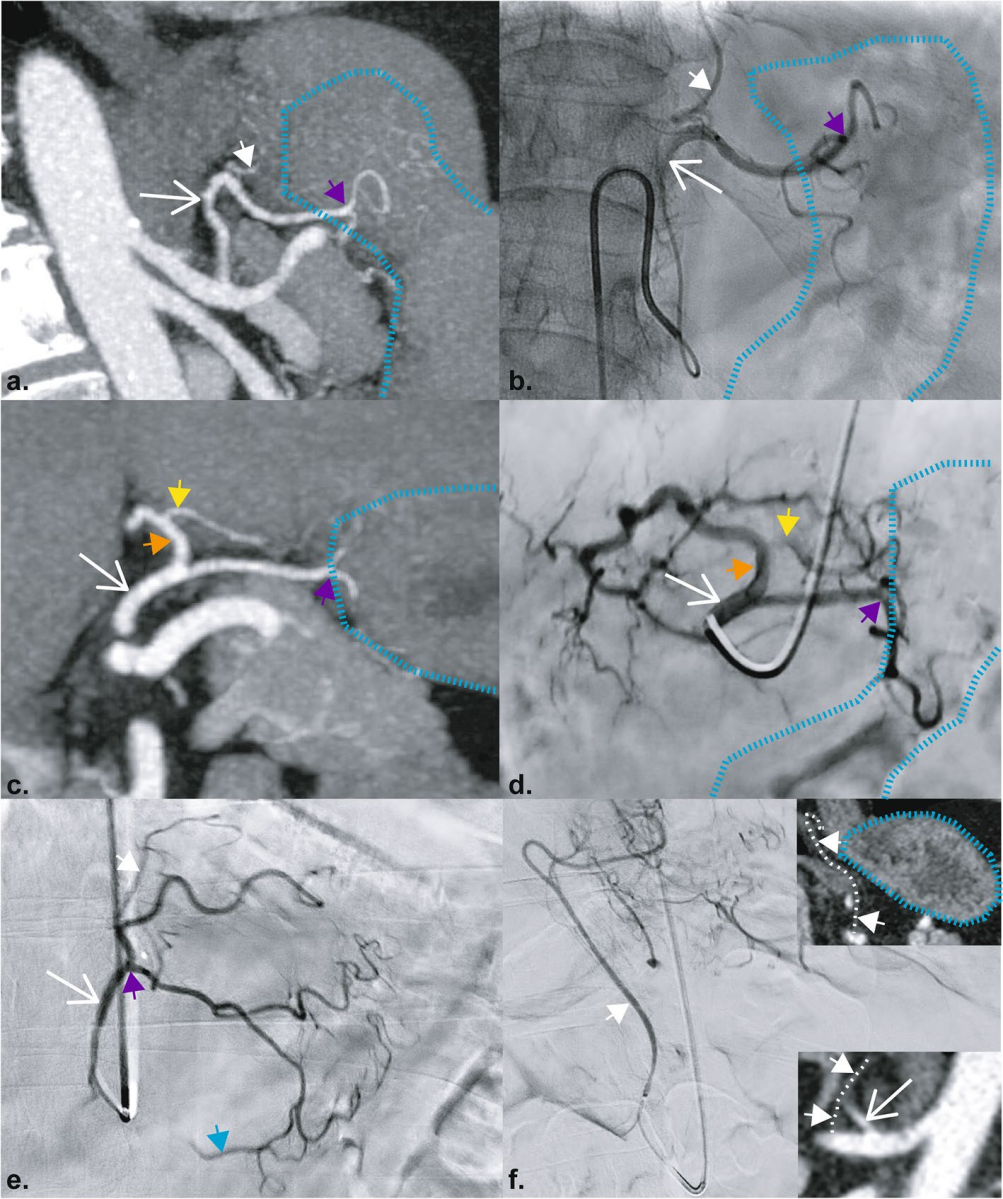
Examples of variant LGA anatomy on CT and catheter angiography. **a** CT angiography of 1b LGA classification. **b** Corresponding catheter angiogram of the same participant. **c** CT angiography of 3e classification of the LGA. There is an accessory left hepatic artery (orange arrow) with an accessory LGA (yellow arrow). **d** corresponding catheter angiogram in the same participant. **e** Catheter angiography of a participant with 2c classification with the GOJ artery arising from the superior branch of the LGA and a large right gastric artery anastomosis with flow reversal (blue arrow). **f** Catheter angiogram of the GOJ artery originating directly from the coeliac trunk. The top insert shows the course of the GOJ artery (white dashed line) in the coronal plane on and the bottom insert shows its origin just distal to the LGA from the coeliac trunk in the sagittal plane. Key: LGA, long white arrow; GOJ artery, short white arrow and white dashed line; accessory LGA, yellow arrow; the bifurcation of the LGA, purple arrow; accessory left hepatic artery, orange arrow; right gastric artery anastomosis, blue arrow; stomach outline, blue dashed line

The branching pattern of the LGA was categorised by factors pertinent to successful embolisation of the gastric fundus without off-target embolisation (Table 5, Fig. 5). Firstly, the LGA was categorised if it provided collateral supply to the left lobe of the liver or a visible anastomosis with the RGA. Secondly, if the LGA had a branch supplying the gastric cardia and or gastro-oesophageal junction/oesophagus (GOJA) and if there was an accessory LGA originating from the replaced/accessory left hepatic artery (Fig. 6). In a single participant the GOJA originated directly from the coeliac trunk distal to the LGA (Fig. 6e).

**Table 5:** Frequency of the different LGA classifications

Classification	Count
**1.**	**44**
a.	10
b.	32
c.	2
**2.**	**26**
a.	7
b.	16
c.	3
**3.**	**13**
a.	1
b.	1
d.	8
e.	1
f.	2
**4.**	**3**
d.	1
g.	2
**Total**	**86**

In seventy-four participants (86%) the main LGA supplying the stomach *i.e.*, excluding branches to other organs and the GOJA, bifurcated to two main branches supplying the stomach, typically in a superior and inferior configuration (Fig. 6). In eleven participants the main LGA trifurcated, while in one participant there was a quadfurcation to four large branches. Where an RGA anastomosis was observed (34%, *n* = 29) this was invariably from the inferior branch of the LGA which traversed inferiorly along the lesser curve supplying the inferior fundus and body.

### Other gastric supply and assessment of fundal arterial supply

The RGEA and short gastric arteries were identified in all ninety participants on CT. The RGA and LGEA were identified in fifty-three (59%) and twenty-eight participants (31%), respectively. The LGEA was defined separately from the short gastric arteries as a dominant gastric branch of the splenic artery with or without an RGEA anastomosis.

In forty-six participants (51%) the LGA was the dominant artery supplying the gastric fundus. The LGEA and short gastric arteries were identified in one participant each as being the sole dominant artery to the gastric fundus. In all other participants a single dominant artery to the fundus was not identified with supply coming from multiple arteries, most commonly shared between the LGA and short gastric arteries (27%, *n* = 24). In three participants the LGA did not contribute significantly to fundal blood supply.

### Inter-observer agreement

95% limits of agreement (LoA) and bias were calculated between readers (Table S1, Fig. S1 and S2). Although absolute variability in diameter measurement decreased with decreasing with vessel diameter, as a proportion of mean vessel diameter variability increased from 27% (aorta), 52% (coeliac trunk) and 89% (LGA). The maximum bias for any measurement was 1.6 ± 1.6 mm (reader 3 vs. 2, AP aorta 3 cm above the coeliac trunk).

There was good agreement between readers for the angles of the coeliac trunk. The mean range of the 95% LoA was 40°(range 19 - 53°) and 27° (range 20 - 35°) for the para-sagittal and axial clockwise angle, respectively. Measurements of the LGA angle were made by readers 1 and 3 with ranges of the 95% LoA of 68° and 70° for the para-sagittal and para-coronal clockwise angle, respectively.

## Discussion

The coeliac trunk and hepatic arterial anatomy has been extensively described with several existing classification systems, with the focus on describing the anatomy relevant to hepatobiliary open surgery [16–18]. However, the gastric arteries have been less extensively studied and the anatomical considerations for interventional radiology are different from open surgery as knowledge of branch origin angles, location and collateral supply are essential to successful case planning. *A priori* knowledge of the vascular anatomy is particularly important in obese patients since the pre- and intra-operative imaging are likely to be suboptimal due to body habitus. For example, the mean clockwise angle of origin of the coeliac trunk in the axial plane in this study was 18° (i.e. to the left of the midline), therefore a perpendicular projection to facilitate catheterisation would be 72° RAO (right anterior oblique) for the supine patient. Alternatively, a screening angle of approximately 20° RAO is sufficient to offset the coeliac trunk and LGA from the spine to improve catheter visualisation and both can usually be catheterised *en face*, often without aortic/coeliac trunk digital subtraction angiography.

The para-sagittal angle that the coeliac trunk originates from the aorta can help inform the optimal catheter tip shape. For example, to access a coeliac trunk originated at the mean angle of 124° from a radial approach, a multipurpose catheter that has a tip length of 2 cm to extend across the diameter of the aorta at an angle of 120° should theoretically be a good choice. For patients with acute angles of ~ 50° a reverse curve catheter e.g., a Simmons 1, may be better suited.

Knowledge of the potential collaterals that may be seen during catheter angiography will help to maximise therapeutic effect while minimising off-target embolisation. Collateral supply from the LGA to the GOJ (78%), left lobe of the liver (19%), and diaphragm (8%) were demonstrated on CT. In participants with paired catheter angiography additional phrenic collaterals arising from the LGA and GOJA were identified that were not visible on CT, a recognised finding in cadaveric studies [19, 20]. Off-target embolisation of these collaterals increases the risk of acute liver injury (hepatic), peri-operative pain (phrenic) ischaemia and perforation (oesophageal/gastric cardia) [21–23].

The stomach has a rich arterial supply from paired and anastomosing gastric and gastroepiploic arteries, posterior gastric and short gastric arteries [24]. LGA-RGA anastomoses are considered ubiquitous in cadaveric studies and the low rate reported here is largely secondary to the failure to identify the small RGA on CT [20]. The LGA was the dominant fundal artery in approximately half of participants, while in the rest fundal supply was shared between two or more arteries. The natural redundancy in arterial supply which acts as a protective mechanism for ischaemic infarction may also be a mechanism for technical failure of embolisation for obesity treatment [25].

Multiple readers were used to minimise single-reader bias and assess reproducibility, which compared favourably to a previous study [26]. Expectedly, there was poorer reproducibility of angle measurements, particularly of the LGA.

This study had several limitations. The inclusion criteria was participation in the EMBIO trial which had a female bias, although no significant difference between sexes were identified for any measurement in this study, contrary to previous studies [27, 28]. The anatomical assessment can be limited in obese patients due to increased noise, particularly when studying small gastric vessels [29]. CT scans were acquired in inspiration and this may increase the coeliac angle. CT also has a tendency to underestimate vessel diameter, which was mitigated by using a 10 mm maximum intensity projection to assess the LGA [30]. There was no ground truth available from surgical or cadaveric examinations and the use of supporting findings from catheter angiography was available in only approximately half of the study cohort.

## Conclusion

This study presents a comprehensive assessment of the angiographic anatomy of the left gastric artery and relevant coeliac trunk anatomy from CT and catheter angiography in obese patients. The findings presented should aid interventional radiologists to perform a safe and effective LGA embolisation for treatment of obesity as well as for upper gastrointestinal bleeding.

## Supplementary Information

Supplementary Material 1.

## Data Availability

The dataset generated and analysed during the current study is available from the UCL Repository, 10.5522/04/28254554.v1.

## Abbreviations

aLGA: Accessory left gastric artery
CHA: Common hepatic artery
CT: Computed tomography
GDA: Gastro-duodenal artery
GOJA: Gastro-oesophageal artery
EMBIO: Embolisation in Obesity double-blinded, placebo-controlled, trial
LGA: Left gastric artery
LGEA: Left gastro-epiploic artery
LHA: Accessory or replaced left hepatic artery
RGA: Right gastric artery
RGEA: Right gastro-epiploic artery
SG: Short gastric arteries
