# Supplementary material for: Computed tomography assessment of the gastric arterial anatomy for embolisation treatment of obesity
Source: CVIR Endovasc. 2025 Oct 16;8:84. doi: 10.1186/s42155-025-00552-z (PMC12528509; doi:10.1186/s42155-025-00552-z)
Supplement: Supplementary file 1 — Supplementary Material 1. [file 42155_2025_552_MOESM1_ESM.docx]

|  | **Reader 1 vs. 2** | | **Reader 1 vs. 3** | | **Reader 3 vs. 2** | |
| --- | --- | --- | --- | --- | --- | --- |
| **Measurement** | **n** | **Bias ± SD (95% LoA)** | **n** | **Bias ± SD (95% LoA)** | **n** | **Bias ± SD (95% LoA)** |
| **Aortic diameter 3 cm above CT** |  |  |  |  |  |  |
| **AP** | 86 | 1.2 ± 1.7 (-2 to 4.5) | 41 | 0.0 ± 0.8 (-1.6 to 1.6) | 37 | 1.6 ± 1.6 (-1.6 to 4.8) |
| **TR** |  |  |  | -0.4 ± 1.0 (-2.4 to 1.7) |  |  |
| **Aortic diameter at CT level** |  |  |  |  |  |  |
| **AP** | 86 | 0.4 ± 1.7 (-2.9 to 3.8) | 41 | 0.1 ± 1.5 (-2.8 to 3) | 37 | 0.4 ± 1.7 (-2.9 to 3.7) |
| **TR** | 86 | 0.0 ± 1.3 (-2.6 to 2.6) | 41 | 0.0 ± 1.1 (-2.1 to 2.1) | 37 | 0.0 ± 1.1 (-2.1 to 2.1) |
| **CT diameter** | 84 | -0.5 ± 0.9 (-2.3 to 1.4) | 41 | -0.2 ± 0.9 (-2.0 to 1.6) | 36 | -0.4 ± 1.0 (-2.3 to 1.5) |
| **LGA diameter** | 84 | 0.2 ± 0.6 (-1 to 1.5) | 41 | -0.3 ± 0.7 (-1.6 to 1.0) | 36 | 0.5 ± 0.7 (-0.8 to 1.8) |
| **CT para-sagittal angle** | 85 | 3.4 ± 11.9 (-19.9 to 26.7) | 41 | 1.8 ± 5.0 (-8.0 to 11.7) | 37 | 0.0 ± 13.5 (-26.5 to 26.4) |
| **CT axial clockwise angle** | 17 | 0.3 ± 9.0 (-17.4 to 17.9) | 41 | 0.1 ± 5.2 (-10.1 to 10.3) |  |  |
| **Aorta to LGA distance** | 78 | 3.1 ± 4.0 (-4.8 to 11.0) | 41 | 1.8 ± 3.9 (-5.9 to 9.4) | 35 | 0.8 ± 3.6 (-6.3 to 7.8) |
| **LGA para-sagittal angle** |  |  | 41 | -2.5 ± 17.4 (-36.6 to 31.7) |  |  |
| **LGA para-coronal clockwise angle** |  |  | 41 | -1.6 ± 17.9 (-36.7 to 33.6) |  |  |

**Table S1. Inter-observer agreement for vessel diameters, angles and distance between the aorta and the LGA ostium.** Abbreviations: AP, anterior-posterior; CT, coeliac trunk; LGA, left gastric artery; TR, transverse. Diameters are measured in millimetres (mm), angles in degrees (°).


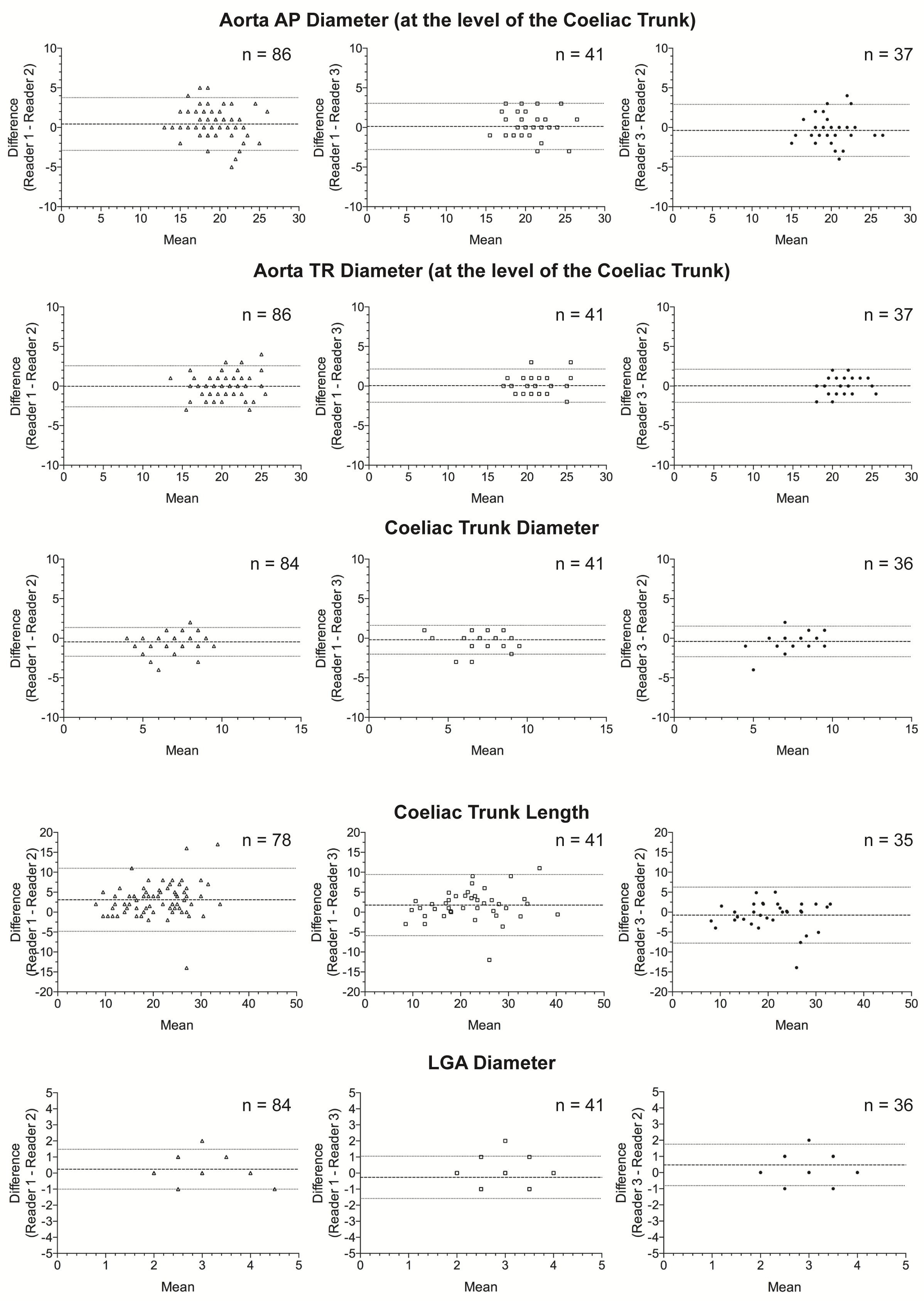


**Figure S1.** Bland-Altman plots showing inter-reader agreement for measurements of vessel diameters and length of the coeliac trunk. Abbreviations: LGA, left gastric artery.


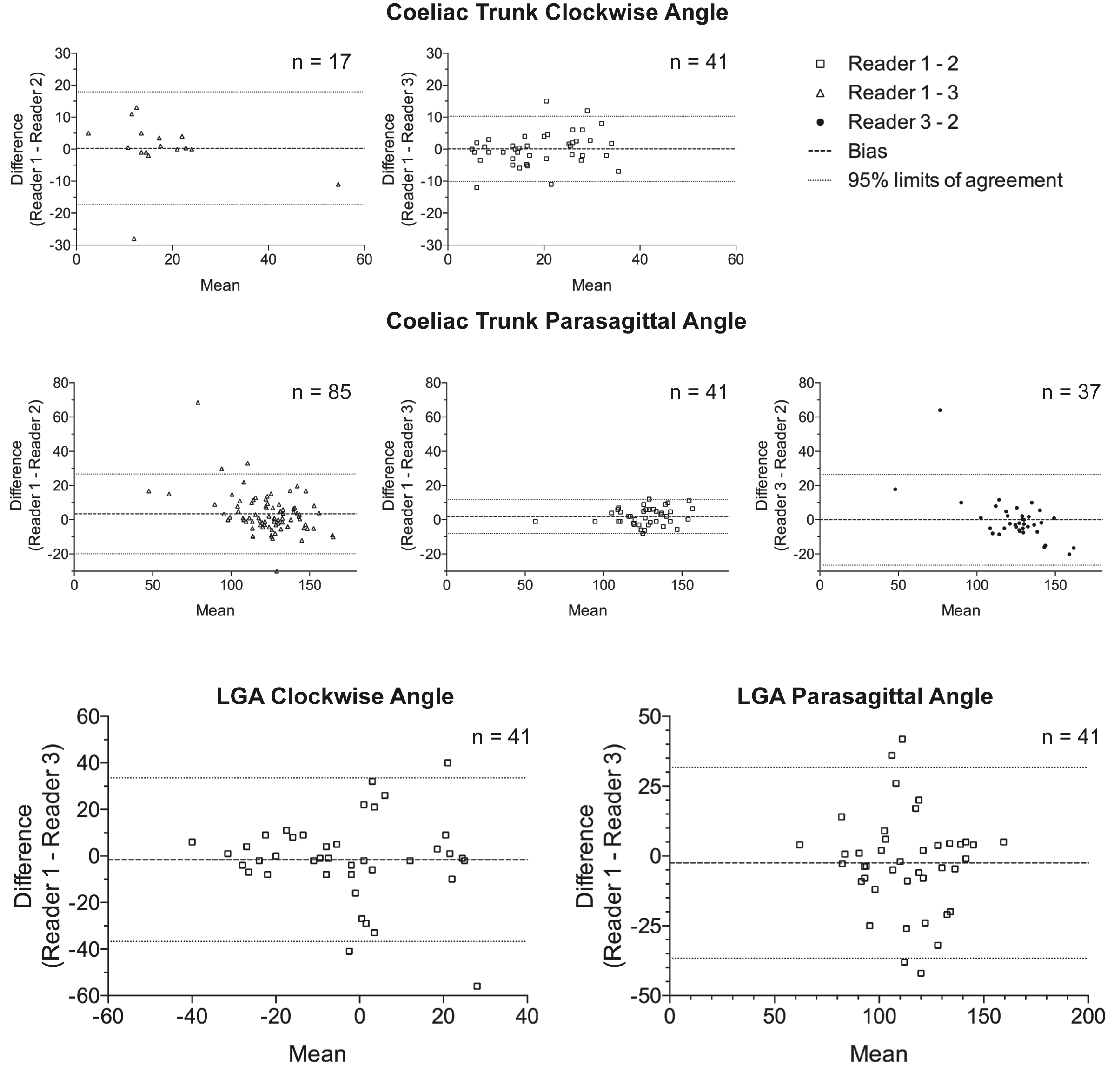
**Figure S2.** Bland-Altman plots showing inter-reader agreement for measurements of the angles of the origin of vessels. Abbreviations: LGA, left gastric artery.


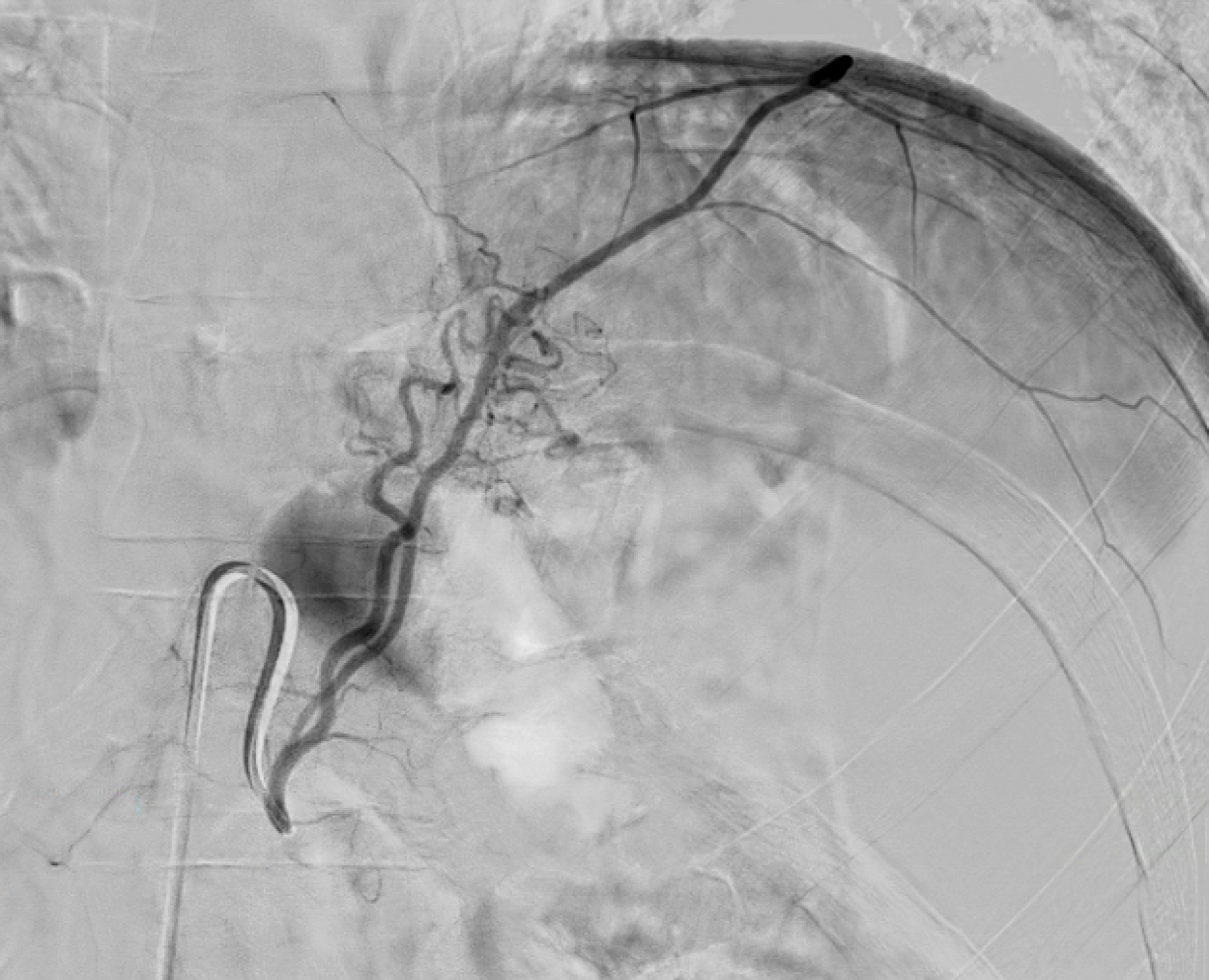


**Figure S3.** Digital subtraction angiogram from the left inferior phrenic artery. This artery can be catheterised accidentally and mimic the left gastric artery.
